# Biocatalyzed Flow Oxidation of Tyrosol to Hydroxytyrosol and Efficient Production of Their Acetate Esters

**DOI:** 10.3390/antiox10071142

**Published:** 2021-07-19

**Authors:** Francesca Annunziata, Martina L. Contente, Cecilia Pinna, Lucia Tamborini, Andrea Pinto

**Affiliations:** 1 Department of Pharmaceutical Sciences, University of Milan, Via Mangiagalli 25, 20133 Milan, Italy; francesca.annunziata@unimi.it; 2 Department of Food, Environmental and Nutritional Sciences, University of Milan, Via Celoria 2, 20133 Milan, Italy; martina.contente@unimi.it (M.L.C.); cecilia.pinna@unimi.it (C.P.); andrea.pinto@unimi.it (A.P.)

**Keywords:** tyrosol, hydroxytyrosol, acetate derivatives, biocatalysis, oxidation, flow chemistry

## Abstract

Tyrosol (Ty) and hydroxytyrosol (HTy) are valuable dietary phenolic compounds present in olive oil and wine, widely used for food, nutraceutical and cosmetic applications. Ty and HTy are endowed with a number of health-related biological activities, including antioxidant, antimicrobial and anti-inflammatory properties. In this work, we developed a sustainable, biocatalyzed flow protocol for the chemo- and regio-selective oxidation of Ty into HTy catalyzed by free tyrosinase from *Agaricus bisporus* in a gas/liquid biphasic system. The aqueous flow stream was then in-line extracted to recirculate the water medium containing the biocatalyst and the excess ascorbic acid, thus improving the cost-efficiency of the process and creating a self-sufficient closed-loop system. The organic layer was purified in-line through a catch-and-release procedure using supported boronic acid that was able to trap HTy and leave the unreacted Ty in solution. Moreover, the acetate derivatives (TyAc and HTyAc) were produced by exploiting a bioreactor packed with an immobilized acyltransferase from *Mycobacterium smegmatis* (MsAcT), able to selectively act on the primary alcohol. Under optimized conditions, high-value HTy was obtained in 75% yield, whereas TyAc and HTyAc were isolated in yields of up to 80% in only 10 min of residence time.

## 1. Introduction

**Figure 1:**
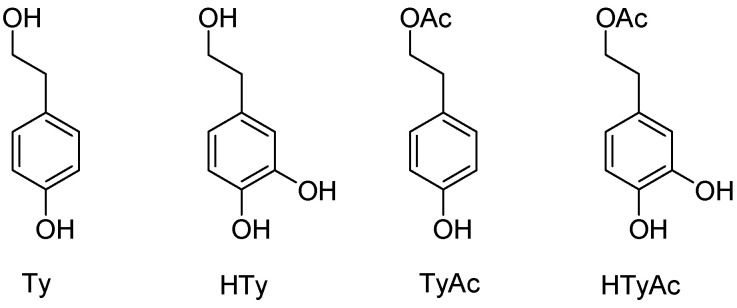
Structures of tyrosol (Ty), hydroxytyrosol (HTy), tyrosol acetate (TyAc) and hydroxytyrosol acetate (HTyAc).

The demand for nontoxic antioxidants that are active in hydrophilic and lipophilic systems has led to the search for natural antioxidants that can be used in oil-based formulas and emulsions. Tyrosol (4-hydroxyphenethyl alcohol, Ty, Figure 1) and hydroxytyrosol [2-(3,4-dihydroxyphenyl)ethanol, HTy, Figure 1] are valuable dietary phenolic compounds present in olive oil and wine and are shown to possess a range of biological effects, including antioxidant, anti-inflammatory, cardioprotective, neuroprotective, anticancer, antidiabetic, and antimicrobial properties [1,2,3,4]. Particularly, HTy is one of the most powerful natural antioxidants due to the presence of the *o*-dihydroxyphenyl moiety [5]. Importantly, toxicological studies demonstrated that HTy is non-genotoxic and non-mutagenic [6,7], raising the possibility of using this compound as a nutraceutical [8,9,10]. It is employed in the food (stabilizer for vegetable oils, beverages, margarines, yogurts, etc.), pharmaceutical (supplements), and cosmetic (sunscreens, lotions, shampoos, deodorizers, etc.) industries. In particular, cosmetic applications of HTy as an anti-aging and anti-inflammatory ingredient have been reported [11]. Considering the industrial applications of HTy, as well as the studies on its biological properties, it is important to have at hand sustainable and efficient synthetic procedures at competitive prices to prepare this compound. Therefore, it is not surprising that many chemical efforts have been made to collect pure HTy, either by synthesis or from natural sources [12]. Particularly, HTy can be obtained from Ty chemically, often using costly reactants [13,14,15]; enzymatically [16]; or from tyrosine through three biocatalyzed steps [17].

Moreover, the acetylation of the primary alcohol of Ty and HTy, to give TyAc and HTyAc (Figure 1), respectively, increases their lipophilicity and lipid solubility, modifying their bioavailability [18], stability [19] and antioxidant effect in cosmetic products and food emulsions [20]. HTyAc was described for the first time in virgin olive oils by Brenes et al. [21]. It has been reported that the acetylation of HTy significantly increases its transport across the small intestinal epithelial cell barrier, offering enhanced bioavailability compared to HTy; consequently, acetylation represents a strategy that can be applied to improve the absorption of polyphenols, thus increasing their potential biological activity [18,22,23,24]. In fact, several studies have reported the antioxidative, anti-inflammatory, neuroprotective and antiarthritic effects of HTyAc [25,26,27,28]. The acetylation reaction can be performed chemically, with acid chlorides or acid anhydrides, but these routes do not meet the requirements necessary for food applications. The use of enzymes in non-aqueous media can overcome this issue. Over the years, the enzymatic esterification of phenolic alcohols has been reported, mainly using lipases [29,30].

In this context, the possibility to have access to pure HTy and its corresponding acetate derivative HTyAc (Figure 1) is very appealing. Therefore, the aim of the present work is the design of sustainable protocols for the biocatalyzed synthesis of high-value HTy starting from easily accessible Ty, and for the obtainment of their acetate derivatives, characterized by increased lipophilicity (Figure 1). To achieve our goal, we exploited the integration of biocatalysis and flow chemistry; these represent ideal partners for accessing novel chemical spaces and define efficient and sustainable synthetic tools with a high level of intensification [31]. In this work, we selected two biocatalysts: a commercially available free tyrosinase from *Agaricus bisporus* for the regioselective oxidation of Ty to HTy; and an immobilized acyltransferase from *Mycobacterium smegmatis* (MsAcT), which was previously reported by us [32,33], for the efficient and selective acetylation of the primary alcohol of Ty and HTy. The use of immobilized biocatalysts represents one of the key tools for increasing sustainability by simplifying the product work-up, allowing easy catalyst separation and reuse; moreover, the use of immobilized enzymes in packed bed reactors under flow conditions ensures high surface-to-volume ratios, thus providing high heat and mass transfer rates, resulting in increased performance.

## 2. Materials and Methods

Reagents and solvents were obtained from commercial suppliers and were used without further purification. NMR spectra were recorded on a Varian Gemini 300 MHz spectrometer using the residual signal of the deuterated solvent as the internal standard. ^1^H chemical shifts (*δ*) are expressed in ppm and coupling constants (*J*) in hertz (Hz). Continuous flow biotransformations were performed using an R2^+^/R4 or a Series E Vapourtec flow reactor equipped with an Omnifit^®^ (Merck, Milan, Italy) glass column (6.6 mm i.d. × 100 mm length), a PTFE coil (10 mL) or a tube-in-tube reactor (15 mL). The temperature sensor sat on the wall of the reactors. Pressure was controlled by using back-pressure regulators. For the in-line extraction, an additional HPLC pump (ThalesNano, Stepbio, Bologna, Italy) was used. In-line liquid/liquid separations were performed using a Zaiput separator. TLC analyses were performed on commercial silica gel 60 F254 aluminium sheets; spots were further evidenced by spraying with a dilute alkaline solution of KMnO_4_. Mushroom tyrosinase from *Agaricus bisporus* (8503 U/mg)*,* sodium phosphate monobasic, sodium phosphate dibasic, ethyl acetate, toluene, sodium sulfate anhydrous, polymer-supported boronic acid and molecular sieves were purchased from Merck (Milan, Italy); tyrosol and ascorbic acid were purchased from Fluorochem (Zentek, Milan, Italy). For MsAcT, protein expression and purification, and free enzyme activity measurements (150 U/mg) were performed following previously reported protocols [34]. One unit (U) of activity is defined as the amount of enzyme which catalyzes the consumption of 1 μmol of substrate per minute. Aldehyde agarose immobilization and immobilized MsAcT activity measurements (120 U/mg) were performed as previously described [32].

### 2.1. Batch Oxidation of Ty by Free Tyrosinase from Agaricus bisporus

The oxidation of Ty into HTy in batch mode was performed according to the method reported by Guazzaroni et al. [35]. Ascorbic acid (1.5 eq.) was added to a solution of Ty (10 mM, 7 mg, 0.05 mmol) in phosphate buffer 0.1 M, pH 7.0 (5 mL). Free tyrosinase (180 µL of a 0.98 mg/mL stock solution in phosphate buffer 0.1 M, pH 7.0) was added and the reaction mixture was stirred at room temperature for 24 h. The aqueous solution was extracted with ethyl acetate, the organic phases were dried over anhydrous Na_2_SO_4_ and the solvent was evaporated. The crude was analyzed by NMR spectroscopy. The obtained conversion was 27%.

### 2.2. Continuous Oxidation of Ty by Free Tyrosinase from Agaricus bisporus Using a Gas/Liquid Biphasic System and In-Line Extraction

Two stock solutions were prepared: (a) a solution of Ty (10 mM, 14 mg, 0.1 mmol) in sodium phosphate buffer 0.1 M, pH 7.0 (10 mL); (b) a solution of tyrosinase 300 U/mL (360 µL of a 0.98 mg/mL stock solution in phosphate buffer 0.1 M, pH 7.0) and ascorbic acid (50 mM, 88 mg, 0.5 mmol) in sodium phosphate buffer 0.1 M pH 7.0 (10 mL). The stock solutions were pumped through the tubular reactor by two HPLC pumps with a total flow rate of 110 μL/min (55 μL/min for each pump). A third peristaltic pump flowed air at 220 μL/min. The three streams (i.e., the two liquid solutions and the air stream) were mixed in a quadruple mixer to form a gas/liquid segmented flow that entered the coil reactor maintained at 28 °C. A 40 psi back-pressure regulator (BPR) was applied to the system. After the residence time (30 min), the exiting flow stream was extracted in-line by adding an inlet of toluene pumped with an external HPLC pump at 333 μL/min. The outlet flow was directed to a liquid/liquid separator and both organic and aqueous phases were collected. The organic phase was dried with anhydrous Na_2_SO_4_ and the solvent evaporated. The crude was analyzed by NMR spectroscopy. The obtained conversion was 78%. Ty: R_f_ (DCM/MeOH 9:1): 0.69; ^1^H NMR (300 MHz, methanol-*d_4_*) δ: 7.05–6.99 (m, 2H), 6.73–6.67 (m, 2H), 3.68 (t, *J* = 7.2, 0.8 Hz, 2H), 2.71 (t, *J* = 7.2 Hz, 2H). HTy: R_f_ (DCM/MeOH 9:1): 0.50; ^1^H NMR (300 MHz, methanol-*d_4_*) δ: 6.71–6.64 (m, 2H), 6.55–6.50 (m, 1H), 3.67 (t, *J* = 7.2 Hz, 2H), 2.66 (t, *J* = 7.2 Hz, 2H).

### 2.3. Continuous Oxidation of Ty by Free Tyrosinase from Agaricus bisporus Using a Tube-in-Tube Reactor and In-Line Extraction

Two stock solutions were prepared: (a) a solution of Ty (10 mM, 14 mg, 0.1 mmol) in sodium phosphate buffer 0.1 M, pH 7.0; (b) a solution of tyrosinase 300 U/mL (360 µL of a 0.98 mg/mL stock solution in phosphate buffer 0.1 M, pH 7.0) and ascorbic acid (50 mM, 88 mg, 0.5 mmol) in sodium phosphate buffer 0.1 M, pH 7.0. The stock solutions were pumped at a total flow rate of 250 μL/min (125 μL/min for each pump) (residence time: 60 min) at 28 °C. The external tube of the reactor was filled with pressurized air (3 bar) and the whole system was pressurized at 40 psi. The exiting flow stream was extracted in-line by adding an inlet of toluene pumped with an external HPLC pump at 250 μL/min and then the biphasic flow stream was separated using an in-line liquid/liquid separator. Both organic and aqueous layers were collected. The aqueous phase, which contained the enzyme and ascorbic acid, was recirculated through the system. The organic phase was dried with anhydrous Na_2_SO_4_ and the solvent was evaporated. The crude was analyzed by NMR spectroscopy. The final conversion was 40%.

### 2.4. Catch-and-Release Procedure

The organic flow stream containing Ty and HTy was flowed through a column (total volume: 3.0 mL) packed with polymer-supported boronic acid (1.0 g, loading 2.5–3.0 mmol/g, 1.5 mL) mixed with an equal volume (1.5 mL) of molecular sieves (4 Å) at 110 °C (BPR 1.0 bar) with a residence time of 1 h (flow rate: 50 μL/min). The exiting flow stream was monitored by TLC and only Ty was present. The organic solvent was collected and evaporated under pressure to recover pure Ty. The release of HTy was performed by flowing 2 N HCl through the column at room temperature with a residence time of 30 min. The solvent was removed under pressure to furnish pure HTy as a yellow oil (75% isolated yield). R_f_ (DCM/MeOH 9:1): 0.50; ^1^H NMR (300 MHz, methanol-*d_4_*) δ: 6.71–6.64 (m, 2H), 6.55–6.50 (m, 1H), 3.67 (t, *J* = 7.2 Hz, 2H), 2.66 (t, *J* = 7.2 Hz, 2H).

### 2.5. Batch Synthesis of TyAc

To a 0.25 M solution of Ty in phosphate buffer 0.1 M, pH 8.0, 200 mg of imm-MsAcT (1 mg/g_agarose,_ 120 U/mg) and 10% v/v of EtOAc (total volume: 1.0 mL) were added. The mixture was left under gentle agitation at 28 °C. After 24 h, the reaction did not reach completion. The reaction mixture was filtered and the aqueous phase was extracted three times with EtOAc. The organic layers were dried over Na_2_SO_4_ and the solvent was evaporated. The crude product was purified by flash chromatography (DCM/MeOH 98:2). TyAc was obtained in 47% yield as a white solid.

### 2.6. Flow Synthesis of TyAc and HTyAc and In-Line Work-Up

A glass column (i.d.: 6.6 mm) was packed with 2.5 g of imm-MsAcT (1 mg/g_agarose_; packed bed reactor volume: 2.0 mL). A 0.25 M Ty or HTy solution in phosphate buffer (0.1 M, pH 8.0, with 10% of DMSO in the case of HTy), and EtOAc were mixed in a T-piece and the resulting segmented flow stream (buffer/EtOAc 7:3) was directed into the reactor column that was kept at 28 °C. The total flow rate was 0.20 mL/min (residence time: 10 min). An inlet of EtOAc (flow rate: 0.2 mL/min) was added to the exiting reaction flow stream using a T-junction and an in-line liquid/liquid separation was performed using a Zaiput liquid/liquid separator. The completion of the reaction was monitored by TLC (DCM/MeOH 9:1). The organic phase was evaporated and purified by flash chromatography (DCM/MeOH 98:2) to yield the desired product. TyAc (yield: 81%): R_f_ (DCM/MeOH 98:2): 0.56; ^1^H NMR (300 MHz, chloroform-*d_3_*) δ: 7.10–7.06 (m, 2H), 6.81–6.74 (m, 2H), 4.24 (t, *J* = 7.1 Hz, 2H), 2.86 (t, *J* = 7.1 HZ, 2H), 2.05 (s, 3H). HTyAc (yield: 75%): R_f_ (DCM/MeOH 98:2): 0.30; ^1^H NMR (300 MHz, chloroform-*d_3_*) δ: 6.82–6.71 (m, 2H), 6.65–6.55 (m, 1H), 4.23 (t, *J* = 7.2 Hz, 2H), 2.81 (t, *J* = 7.2 Hz, 2H), 2.05 (s, 3H).

## 3. Results and Discussion

The first step was the oxidation of Ty by free tyrosinase from *Agaricus bisporus* in the presence of oxygen and ascorbic acid. Tyrosinase (EC 1.14.18.1) is a polyphenol-oxidase that catalyzes the hydroxylation of monophenols to *o*-diphenols and the oxidation of *o*-diphenols to *o*-quinones. The addition of ascorbic acid results in the increased accumulation of catechol, reducing quinones formed by tyrosinase activity back to catechol [36].

To guarantee the oxygen supply, two technical solutions have been evaluated: the use of a tube-in-tube reactor, specifically designed for the execution of reactions involving the presence of a gas reagent [37]; and a segmented air/liquid flow stream in a tubular PTFE reactor. In this last case, flow-based reactors may offer advantages when performing multiphase reactions, including gas/liquid or liquid/liquid reactions, due to facilitated mass transfer and increased interfacial area for the exchange of chemical species [38,39,40,41].

**Scheme 1:**
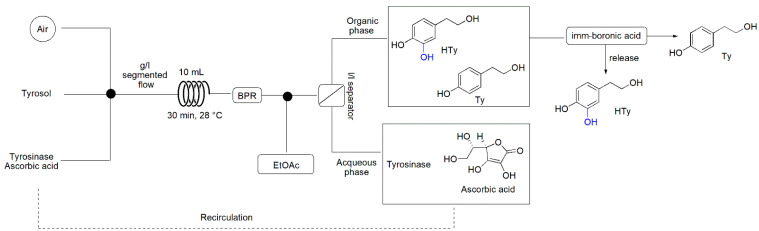
Optimized flow reactor configuration for the bio-oxidation of Ty to HTy and in-line purification. The solutions of Ty and tyrosinase with ascorbic acid were prepared in sodium phosphate buffer 0.1 M, pH 7.0. BPR: 40 psi.

The first experiments were performed using reaction conditions (i.e., substrate concentration, stoichiometry, solvent and temperature) reported for the batch reaction [35]. The conversion obtained by flowing the solutions of the starting materials without a specific inlet of air brought a very low conversion (i.e., 25% molar conversion in 60 min of residence time, Table 1, Entry 1), even when saturating the stock solutions. Using a tube-in-tube reactor, a 40% conversion was achieved (Table 1, Entry 2). The best configuration was obtained by adding an inlet of air, forming an air/liquid biphasic system (Scheme 1); in this way, the conversion increased to 47% (Table 1, Entry 3). An increase in the stoichiometric ratio in favor of ascorbic acid (from 1:2 to 1:5) avoided the formation of overoxidized by-products, resulting in a cleaner reaction with similar conversion (i.e., 48%, Table 1, Entry 4). A reduction in the substrate concentration (from 20 mM to 10 mM) allowed the achievement of 78% conversion in 30 min of residence time (Table 1, Entry 6), which was 2.9-fold the one obtained in batch in 24 h. The higher reaction performance obtained in flow conditions can be explained considering the more efficient mixing and mass transfer in the biphasic system, the better control of reaction parameters (e.g., residence time, temperature) and the circumvention of some critical issues of batch biotransformations such as substrate/product inhibition effects.

The aqueous flow stream was then extracted in-line by adding an inlet of toluene. The two phases were separated using a liquid/liquid separator to recirculate the wastewater containing the biocatalyst and the excess ascorbic acid, thus creating a self-sufficient closed-loop system with improved efficiency. The aqueous phase was recirculated three times with only a slight reduction in the conversion (cycle 1: 78% m.c.; cycle 2: 74% m.c.; cycle 3: 69% m.c.). After the third cycle, a significant reduction in the conversion (56%) was observed, even when adding fresh ascorbic acid to the reservoir solution. Moreover, the organic phase containing HTy and unreacted Ty was purified in-line thanks to a catch-and-release procedure. This strategy, involving supported boronic acids able to trap HTy through the formation of a cyclic borate with the catechol group, leaves the unreacted Ty in the exiting flow stream. The HTy was then released using an acidic solution (2 N HCl). 

**Scheme 2:**
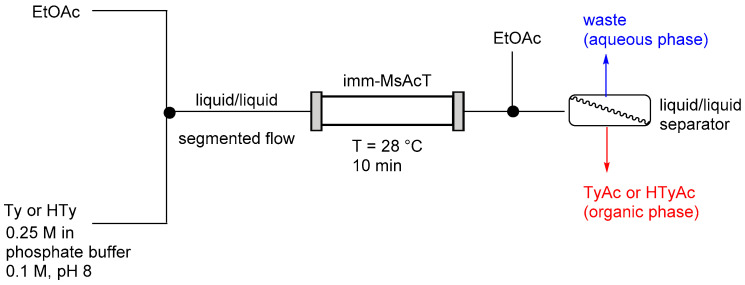
Reactor configuration for the flow-based acylation of Ty and HTy. Column reactor i.d. = 6.6 mm; bioreactor volume = 2.0 mL.

To obtain the corresponding acetate derivatives, a bioreactor packed with an immobilized acyltransferase from *Mycobacterium smegmatis* (MsAcT) was used (Scheme 2). MsAcT shows a characteristic hydrophobic tunnel leading to the active site that can disfavor the ingress of water, thus promoting ester formation over hydrolysis also in aqueous media. The system exploited the covalent immobilization of MsAcT onto agarose beads, increasing the robustness and longevity of the immobilized biocatalyst (enzyme loading 1 mg g_matrix_^−1^). The inlet system was composed with an aqueous solution of Ty or HTy (0.25 M) and an organic phase (pure EtOAc as acetyl donor); the two phases were mixed in a T-piece to form a liquid/liquid heterogeneous segmented flow stream before entering the column. Reactions were performed at 28 °C in phosphate buffer (0.1 M, pH 8.0). Connected software was used to realize an automated process for the collection of the product at the steady state.

Notably, primary alcohol acetylation occurred with complete chemoselectivity, since no reaction involving the phenolic OH was observed, demonstrating that our process is a clean, mild and virtually zero-waste procedure. Full conversion of Ty was achieved after 10 min of residence time using a 7:3 ratio of substrate solution/acetyl donor (EtOAc). A noteworthy observation was that the batch biotransformation did not go to completion in 24 h. An inlet of EtOAc and an in-line liquid/liquid separator were introduced downstream of the process (Scheme 2) for the collection of the organic/aqueous phase. The pure TyAc was obtained by flash chromatography (81% isolated yield). Under the same conditions, HTyAc was isolated in 75% yield. Immobilization coupled with continuous removal of the products gave high stability to the biocatalyst under operating conditions. Moreover, the use of a flow environment associated with a two-liquid phase system avoids the formation of emulsions often present under conventional stirring.

## 4. Conclusions

HTy is a powerful antioxidant with a number of beneficial biological effects and therefore promises to soon become a staple in natural health care. In this work, we developed an environmentally friendly biocatalyzed flow synthesis of HTy using a mushroom tyrosinase and a gas/liquid biphasic system. The precursor Ty is cheaper than both 3,4-dihydroxyphenylacetic acid and oleuropein, which can be used as alternative starting materials for HTy obtainment. The commercial mushroom tyrosinase is rather expensive, but its reutilization by a closed loop system can reduce the overall process cost. Thanks to this automated, self-sustained setup, HTy was obtained with an isolated yield of 75%, which was much higher than the one obtained in batch. The acetate esters TyAc and HTyAc were then efficiently prepared exploiting a bioreactor packed with the immobilized MsAcT, leading to fast reaction times (10 min) and avoiding enzyme destabilization for the formation of the ethanol by-product. It is noteworthy that starting from natural substrates, biocatalytic approaches guarantee the commercialization of the final products as natural too.

## Figures and Tables

**Table 1:** Optimization of reaction parameters.

Entry	Ty (mM)	Ascorbic Acid (mM)	Residence Time (min)	m.c. (%)
1 ^a^	20	40	60	25
2 ^b^	20	40	60	40
3 ^c^	20	40	60	47
4 ^c^	20	100	60	48
5 ^c^	10	50	60	76
6 ^c^	10	50	30	78
7 ^c^	10	50	15	50

The solutions of Ty and ascorbic acid have been prepared in sodium phosphate buffer 0.1 M, pH 7.0. The free tyrosinase was added to the ascorbic acid solution (300 U/mL). The reactions have been performed at 28 °C. Conversions have been determined by ^1^H NMR after in-line extraction with toluene, phase separation and evaporation of the solvent, considering diagnostic signal at 7.05–6.99 for Ty and signal at 6.71–6.64 for HTy. ^a^ Reactor: 10 mL PTFE coil; ^b^ reactor: 15 mL tube-in-tube reactor; ^c^ reactor: 10 mL PTFE coil; air/liquid segmented flow; m.c. = molar conversion.

## Data Availability

Data is contained within the article.
